# Characterisation of the p53-Mediated Cellular Responses Evoked in Primary Mouse Cells Following Exposure to Ultraviolet Radiation

**DOI:** 10.1371/journal.pone.0075800

**Published:** 2013-09-30

**Authors:** Gillian D. McFeat, Sarah L. Allinson, Trevor J. McMillan

**Affiliations:** Division of Biomedical and Life Sciences, Faculty of Health and Medicine, Lancaster University, Lancaster, United Kingdom; German Cancer Research Center, Germany

## Abstract

Exposure to ultraviolet (UV) light can cause significant damage to mammalian cells and, although the spectrum of damage produced varies with the wavelength of UV, all parts of the UV spectrum are recognised as being detrimental to human health. Characterising the cellular response to different wavelengths of UV therefore remains an important aim so that risks and their moderation can be evaluated, in particular in relation to the initiation of skin cancer. The p53 tumour suppressor protein is central to the cellular response that protects the genome from damage by external agents such as UV, thus reducing the risk of tumorigenesis. In response to a variety of DNA damaging agents including UV light, wild-type p53 plays a role in mediating cell-cycle arrest, facilitating apoptosis and stimulating repair processes, all of which prevent the propagation of potentially mutagenic defects. In this study we examined the induction of p53 protein and its influence on the survival of primary mouse fibroblasts exposed to different wavelengths of UV light. UVC was found to elevate p53 protein and its sequence specific DNA binding capacity. Unexpectedly, UVA treatment failed to induce p53 protein accumulation or sequence specific DNA binding. Despite this, UVA exposure of wild-type cells induced a p53 dependent G1 cell cycle arrest followed by a wave of p53 dependent apoptosis, peaking 12 hours post-insult. Thus, it is demonstrated that the elements of the p53 cellular response evoked by exposure to UV radiation are wavelength dependent. Furthermore, the interrelationship between various endpoints is complex and not easily predictable. This has important implications not only for understanding the mode of action of p53 but also for the use of molecular endpoints in quantifying exposure to different wavelengths of UV in the context of human health protection.

## Introduction

Ultraviolet (UV) radiation constitutes only a fraction of the radiation emitted by the sun but it has a large impact on biological activity. UV has become the subject of increasing concern and investigation due to depletion of the ozone layer and the continuing increase in the incidence of skin cancer. The UV component of sunlight incident on the Earth's surface can be broadly divided into UVA (320–400 nm, approximately 90%) and UVB (290–320 nm, approximately 5%) wavebands. UVC (200–290 nm) is largely prevented from reaching the surface of the Earth by its efficient absorption by ozone in the atmosphere. It is well documented that both the UVB and UVC wavelengths are strongly absorbed by DNA leading mainly to the formation of cyclobutane pyrimidine dimers (CPDs) and 6-4 photoproducts (6-4 PP) [1]. In contrast, UVA is only weakly absorbed by DNA and exerts its genotoxic effects presumably through sensitiser radicals and reactive oxygen species (ROS) [2], [3]. Therefore the nature and amount of damage induced by UV radiation is wavelength dependent although it is increasingly evident that both UVB and UVA can have significant deleterious effects on human health [4], [5]. Recently, UV radiation (UVB and UVA) has been classified as a Class I carcinogen by the International Agency for the Research on Cancer [6].

The p53 tumour suppressor protein has long been recognised as being a critical controller of the response of cells to a variety of stresses, including various types of DNA damage [7], [8]. The p53 protein is constitutively expressed in almost all cell types and is normally maintained at a low level. Cellular damage can trigger post-translational modification and association of p53 with other proteins leading to an increase in p53 protein levels and/or transcriptional activities (reviewed in [9]). The genes affected by this participate in critical cellular processes such as DNA repair (reviewed in [10], cell cycle control [11], replicative senescence [12] and programmed cell death [13] which places p53 centrally in the response to DNA damage.

The influence of UV on the p53 response [14]–[16] is important because of the need to understand the action of such an important carcinogen and because damage responses have been proposed as important markers of UV exposure in the context of approaches to protect skin from the harmful effects of the sun. However, the inter-relationship between UV and p53 responses are not clear because of variations in wavelength and dosimetry in previous studies. The objective of this study was therefore to examine the induction of p53 in primary mouse fibroblasts following exposure to different wavelengths of UV radiation and to investigate the impact on downstream events including cell cycle arrest and apoptosis.

## Materials and Methods

### Cell Lines

P53+/+ mouse embryonic fibroblasts (MEFs) were originally obtained from Tyler Jacks (Massachusetts Institute of Technology, Cambridge, MA; [17]). MEFs were grown as a monolayer in Dulbecco's Modified Eagle Medium (Gibco) supplemented with 10% calf serum, penicillin (100 U mL^−1^), streptomycin (5 U mL^−1^) and glutamine (0.2 mM). Cells were maintained at 37°C using humidified air supplemented with 5% CO_2_. Cells were periodically checked for mycoplasma infection and found to be negative.

### UV treatment

Cells were grown to confluency prior to seeding at a density of 2×10^5^ cells in 60 mm dishes. Cells were treated 6 hours after plating at which point approximately 90% of cells were in G1 phase. Exponentially growing cells were treated 18 hours after release when the majority of cells were in S phase. Prior to irradiation medium was removed and cells were washed in phosphate buffered saline (PBS). Cells were irradiated at 4°C in 1 mL of cold PBS using the following UV sources: UVC was provided by a germicidal lamp (Gallenkamp) with peak output at 254 nm. UVB was supplied by four Philips TL40 tubes (Starna Ltd, Romford, U.K.) and wavelengths below 292 nm were eliminated by 100 µm thick cellulose diacetate sheets (Clarifoil, Courtaulds Ltd, Derby, U.K.). The irradiance ranged from approximately 290 to 370 nm and peak output was at 315 nm. UVA was provided by four fluorescent ‘blacklight’ tubes (Philips TLD 36/08, Starna Ltd, Romford, U.K.). Contaminating wavelengths in the UVB and UVC were eliminated by covering petri dishes with polyester (No. 130 clear, Lee Filters, Hampshire, U.K., spectrally equivalent to Mylar). The irradiance ranged from approximately 350 to 400 nm and peak output was at 365 nm. Radiation measurements were made using a double monochromator spectroradiometer (Model SR991-PC, Macam Photometrics, Livingston, U.K.). Unless otherwise indicated, cells were irradiated in dishes on a brass cooling plate, to maintain the irradiation temperature below 8°C and prevent DNA repair taking place during treatment. Following irradiation cells were further cultured in the original medium (that had been taken off the cells before treatment) for the indicated periods of time prior to being subjected to analysis using the following protocols.

### Clonogenic Assay

Clonogenic assays were performed by seeding a known number of cells into 60-mm tissue culture dishes in 5 mL of complete media. After a 6 hour incubation at 37°C, 5% CO_2_ the medium was removed and replaced with PBS. The dishes were then drug exposed or irradiated under the appropriate UV tubes. Following treatment the medium was replaced and the dishes re-incubated for 10 to 14 days. Colonies of at least 50 cells were scored visually after fixation with 70% ethanol and staining with Giemsa (1∶20 dilution in dH_2_O). Each experiment was performed a minimum of three times using triplicate cultures for each dose. IC_37_ values were determined using log-linear interpolation. The surviving fraction (SF) was determined as the number of viable colonies divided by the number of cells seeded and corrected by the observed plating efficiency (PE) for the cell line.

### Bivariate Cell Cycle Analysis by Flow Cytometry

Cells were pulse-labelled with 10 µM bromodeoxyuridine (BrdU) (Sigma) for 50 minutes, prior to fixation as described above. Nuclei were then extracted by protease treatment (0.16 mg mL^−2^ pepsin, 1.6 M HCl) for 30 minutes at 37°C. The acid was neutralised with 1 M Tris and after several washes in chilled PBS. BrdU was detected using a fluorescein isothiocyanate (FITC)-conjugated anti BrdU antibody (10 µg mL^−1^, Becton-Dickinson) diluted in PBS with 0.5% Tween-20 and 1% bovine serum albumin (BSA). Total DNA content was determined by propidium iodide (PI) staining. Cells were incubated for 10 minutes at 37°C with RNase A (100 µg mL^−1^) and PI (5 µg mL^−1^). Cell cycle analysis was carried out immediately with a fluorescence-activated cell sorter (FACScalibur; Becton-Dickinson).

### Immunofluorescent Staining with Anti-p53 Antibody and Detection by Flow Cytometry

All procedures were carried out at 4°C unless otherwise indicated. Following UV treatment cells were detached in trypsin and washed in PBS. Cells were centrifuged and fixed by the dropwise addition of 1 mL of ice-cold methanol. Samples were stored at −20°C for at least 12 hours. After fixation cells were pelleted, washed once with PBS and once with PBS, 0.1% Tween-20 and 0.1% BSA (PBS-T-B). Cells were incubated with a 1∶50 dilution of mouse monoclonal anti-p53 antibody Ab-1 (Calbiochem) for 20 minutes at room temperature, washed three times in PBS-T-B and resuspended in a 1∶100 dilution of FITC conjugated sheep anti-mouse IgG antibody (Calbiochem). After three further washes in PBS-T-B cells were analysed using a fluorescence activated cell sorter (FACScalibur; Becton-Dickinson).

### Detection of p53 by Western Blotting

Whole cell extracts were obtained by scraping cells in 2× lysis buffer (62.5 mM Tris-HCl (pH 6.8 @ 25°C), 2% w/v SDS, 10% gycerol, 0.1% w/v bromophenol blue and 50 mM DTT). Lysates were boiled for 3 minutes, cooled on ice and centrifuged prior to storage at −20°C. Protein concentration of lysates was determined by the Bio-Rad protein assay, according to the manufacturers guidelines (Bio-Rad). Lysates (8–12 mg of total cellular protein and 20 ηg purified control p53 protein) were analysed by 12% sodium dodecyl sulphate-polyacrylamide gel electrophoresis (SDS-PAGE). Proteins were transferred onto nitrocellulose membrane and probed with rabbit polyclonal anti-p53 antibody, FL-393 (1∶400 dilution, Santa Cruz), followed by peroxidase conjugated anti-rabbit antibody (1∶2000 dilution, Pierce). Proteins were detected using enhanced chemiluminescence (Amersham) according to the manufacturer's recommendations. The membrane was exposed to Kodak X-Omat film and the relative amounts of detected proteins were determined by densitometry using image analysis software. Blots were reprobed with anti-actin antibody (1∶500 dilution, Autogen Bioclear) to confirm equal protein loading.

### Annexin V-FITC Detection of Apoptosis

The media from dishes of treated and untreated cells was transferred to 15 mL conical tubes. The cells were washed in PBS and incubated in trypsin until the cells just began to detach. The appropriate medium was returned to each dish, the cells were resuspended and then centrifuged to remove trypsin and prevent cell damage during processing. Approximately 5×10^5^ cells were washed once carefully with PBS and stained with 200 µg mL^−1^ Annexin-V-FITC (Calbiochem) in staining solution (5×: 10 mM Hepes pH 7.4, 150 mM NaCl, 2.5 mM CaCl_2_, 1 mM MgCl_2_, 4% BSA) for 15 minutes in the dark. PI (50 µg mL^−1^) (Sigma) was added and cells were immediately analysed by flow cytometry. Controls consisting of cells only to determine autofluorescence, PI only and annexin only were essential in order to compensate for any overlap between these two signals. Viable cells do not bind PI or annexin V-FITC, early apoptotic cells bind annexin V-FITC due to membrane phospholipidphosphatidylserine (PS) on their surface but their membranes remain intact and they exclude PI. Late apoptotic or necrotic cells are both annexin V-FITC and PI positive.

### P53 Electrophoretic Mobility Shift Assay (EMSA)

Following irradiation, approximately 5×10^5^ cells were scraped into 1.5 mL of cold PBS in a siliconised microfuge tube and nuclear extracts were prepared as described previously [18]. Double-stranded oligonucleotides representing the consensus p53 binding site in the p21^waf1^ gene promoter were purchased as part of a murine p53 NUSHIFT kit (Geneka Biotechnology, Inc.).

Wild-type p53 oligonucleotide:

5′ AGC T GG ACA TGC CCG GGC ATG TC C 3′ [consensus binding sequence]

3′ TCG A CC TGT ACG GGC CCG TAC AG G 5′

Radiolabelling of oligonucleotide probe was performed using a 5′ ^32^P end-labelling kit (Amersham Pharmacia Biotech). Labelled probe was purified on a MicroSpin G25 column. Binding reaction mixtures contained 5 µg of nuclear extract, 4 µL of 2× binding buffer (40 mM HEPES-KOH pH 7.9, 50 mM KCl, 0.2 mM EDTA, 20% glycerol, 4 mM MgCl, 1 mM DTT, 0.05% NP-40, 4 mM spermidine (Sigma), 100 ng poly(dI-dC) (Amersham Pharmacia Biotech) and if indicated 2 µL of monoclonal anti-p53 antibody in a final volume of 20 µL. Binding reaction mixtures were incubated at room temperature for 20 minutes, 0.2 ηg of labelled oligonucleotide probe was added and incubation was continued for a further 20 minutes at room temperature. The entire contents of each reaction tube were loaded onto a 5% non-denaturing polyacrylamide (19∶1) gel containing 5% glycerol. The gel was precooled to 4°C in 1× Tris-Glycine electrophoresis (TGE) buffer (5× TGE: pH 8.5). The gel was run at 4°C in order to avoid heating which may disturb protein-DNA complexes. Migration of the gel was monitored by placing loading buffer (bromophenol blue) into a blank well. The gel was dried on a vacuum gel drier and exposed to Kodak X-Omat film with two X-ray intensifying screens for approximately 2 to 4 hours at −80°C.

The data represented in this paper are available linked to the manuscript at http://www.research.lancs.ac.uk/portal/.

## Results

### Cytotoxicity of UV Radiation

Survival of UV-irradiated MEFs was quantified by clonogenic assay and Table 1 shows the dose of UV radiation required to reduce survival by 37%. As expected UVA was much less toxic than UVB and UVC but the dose range used is still within that encountered environmentally [19].

**Table 1:** Sensitivity of mouse embryonic fibroblasts to ultraviolet radiation as determined by clonogenic assay.

Wavelength	D_37_ (Jm^−2)^
**UVC**	5.4±0.2
**UVB**	483.3±46.0
**UVA**	8.87×10^4^±9.6×10^3^

The D_37_ value shown is the dose of ultraviolet radiation yielding 37% cell survival (mean of 3 or more independent experiments ± standard deviations).

UVA = ultraviolet A; UVB = ultraviolet B; UVC = ultraviolet C.

### Effect of UV Irradiation on P53 Protein Accumulation

**Figure 1:**
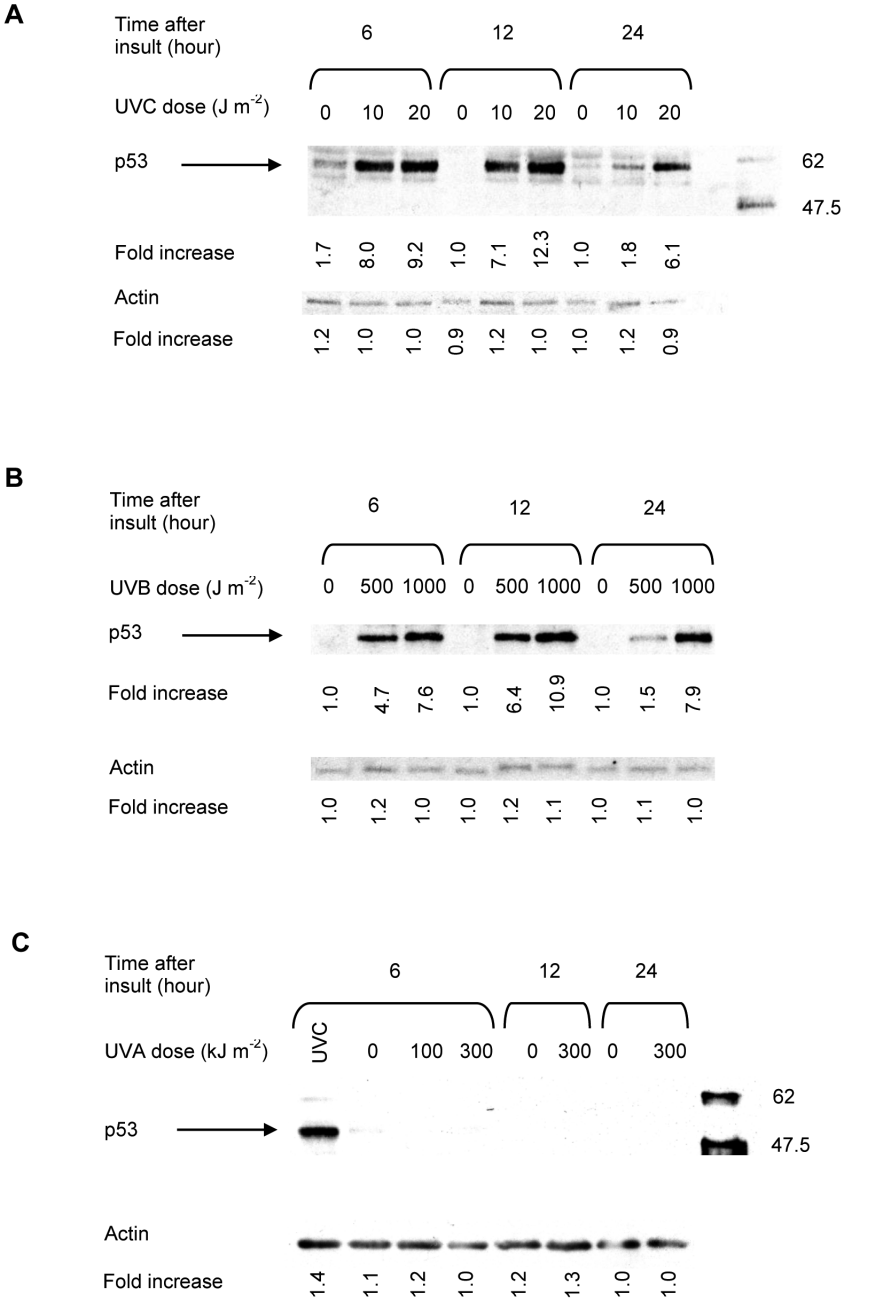
Representative western blot analyses of irradiated fibroblasts treated in G1 phase of the cell cycle. The fold increase in p53 protein accumulation was determined by densitometry. The density of the protein band in untreated samples was considered to be 1.0. Each value is the mean ± S.E.M. of at least of three independent experiments. S.E.M. = standard error of the mean; UVA = ultraviolet A; UVC = ultraviolet C.

Stabilisation of p53 protein in cells is considered to be a general protective response elicited by DNA damage [20]. In agreement with previous reports, exposure of MEFs to UVC resulted in a dose- and time-dependent elevation in p53 protein levels. Maximum accumulation of p53 (approximately 12-fold over mock irradiated controls) was observed with UVC doses between 10 and 20 J m^−2^ (Figure 1A) within 6 to 12 h of treatment. Accumulation was sustained for 24 hours but declined thereafter (Figure 1A and data not shown). Similar results were observed following UVB exposure of cells (Figure 1B). Conversely, MEFs failed to exhibit an accumulation of p53 protein following UVA exposure, regardless of the UVA doses used or the post-exposure times examined (Figure 1C).

**Figure 2:**
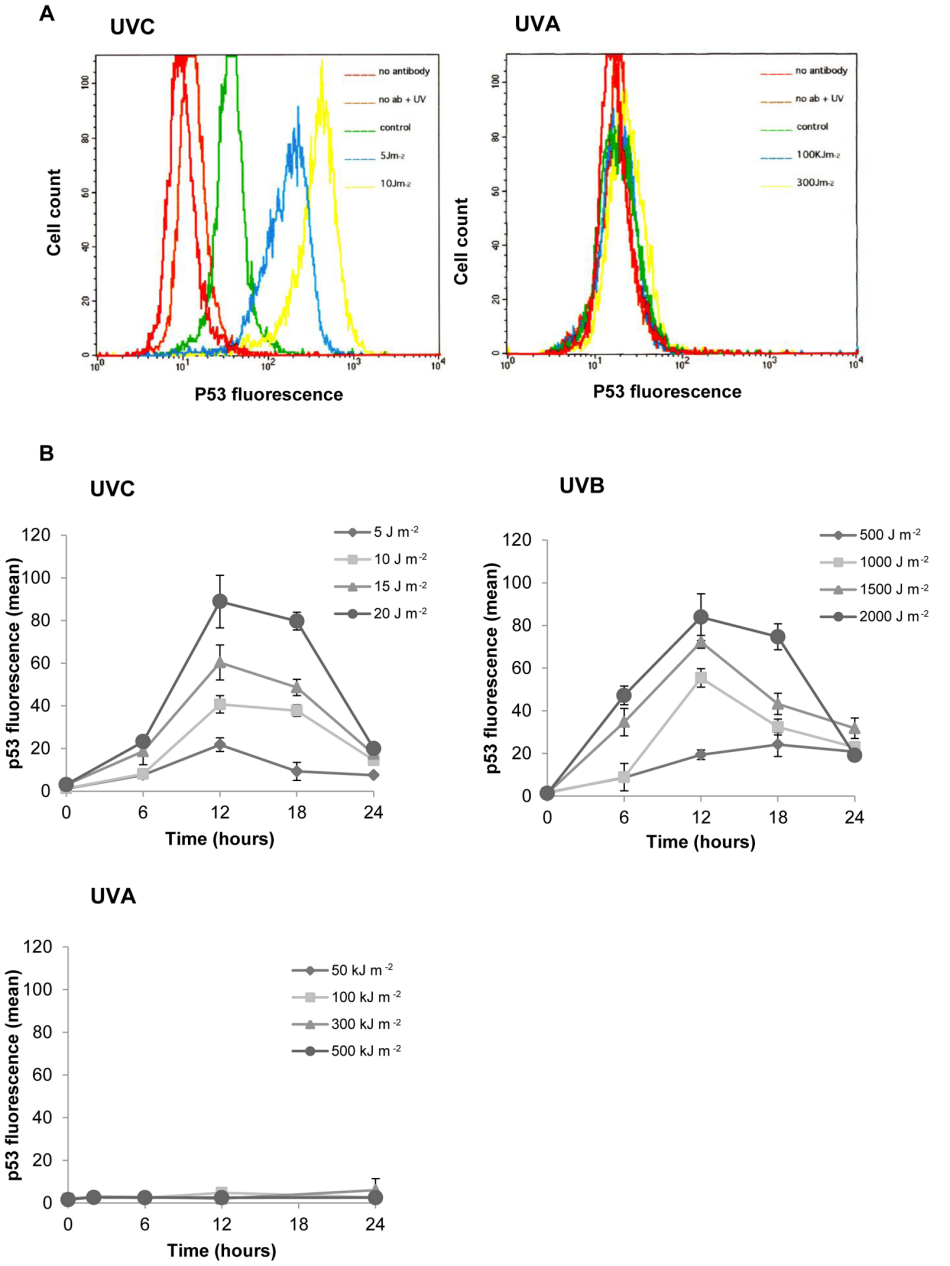
Kinetics and dose dependence of p53 accumulation as determined by fluorescence-activated cell sorting. Figure 2A. Representative flow cytograms of fibroblasts, stained for p53 with a fluorescein isothiocyanate-conjugated antibody. Cells were exposed to ultraviolet radiation in the exponential phase of growth and assayed 6 hours post insult. Figure 2B. P53 accumulation in G1 irradiated fibroblasts in response to ultraviolet radiation. Each data point represents the mean ± S.E.M. for at least 3 independent experiments. S.E.M = Standard error of the mean.

A more sensitive method of p53 protein detection was subsequently employed to confirm the lack of p53 accumulation in response to UVA radiation. Agrawal *et al.* [21] demonstrated the use of flow cytometry as a tool for the measurement of low levels of p53 protein. Example flow cytograms are shown in Figure 2A. Cells were irradiated with increasing doses of UVC, UVB or UVA and incubated for 6 hours prior to flow cytometric analysis. A FITC-conjugated antibody was used to detect p53 and is represented on the x-axis. The mean channel value was taken as the mean number of antibody binding sites and indirectly as the mean number of p53 molecules contained in the cells. Mock-irradiated and irradiated samples stained with secondary antibody alone or no antibody at all served as controls for cellular autofluorescence. Any autofluorescence detected was subtracted from the corresponding values obtained for p53 stained cells to give a corrected mean fluorescence intensity of p53 staining. The relative p53 protein levels in control mock-irradiated samples were subtracted from the corresponding UV-irradiated samples.

As revealed in Figure 2B, a gradual but significant, dose dependent increase in p53 levels was observed in G1 UVB- and UVC-irradiated fibroblasts, peaking at 12 hours and returning to control levels by 24 hours. The extent of p53 accumulation was very similar after exposure of cells to equitoxic doses of UVC and UVB radiation. No increase in the cellular concentration of p53 was detected in response to UVA irradiation of murine fibroblasts. These results obtained using flow cytometry were concordant with those obtained using western blotting to evaluate p53 accumulation.

### The Induction of Transcriptionally Active P53 Protein by DNA Damage

**Figure 3:**
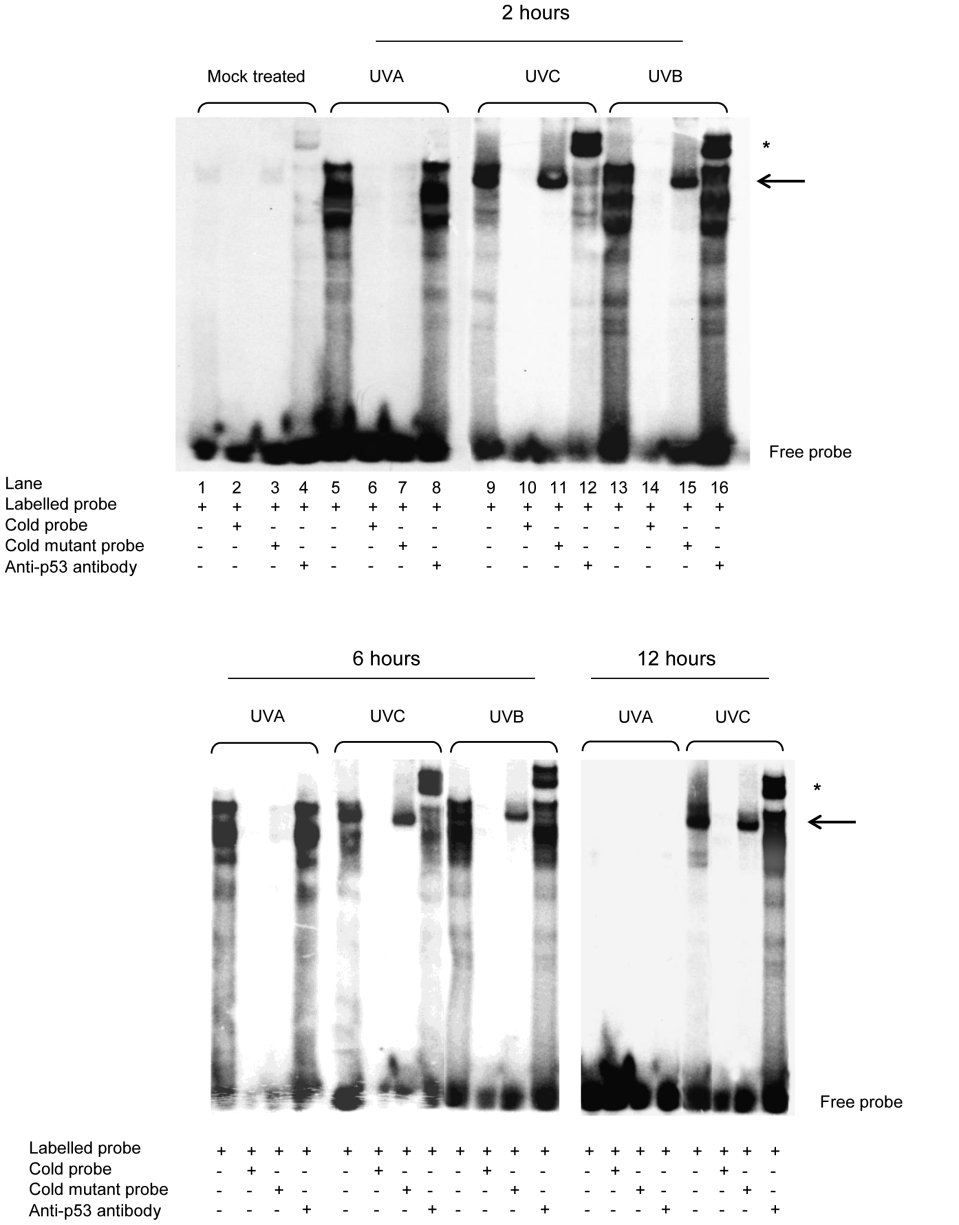
DNA binding by p53 protein after ultraviolet irradiation. Electrophoretic mobility shift assays were performed using a labelled p21^WAF1^ oligonucleotide probe in mouse embryonic fibroblasts. The solid arrow indicates p53-DNA binding complexes; the asterisk indicates supershift of p53-DNA binding complexes with anti-p53 antibody.

The transcriptional activity of p53 has previously been dissociated from its stabilisation following exposure to UV radiation [22]. In light of the lack of p53 protein accumulation in response to UVA irradiation of mouse fibroblasts, the capacity of UV-damage induced p53 protein to bind sequence specific motifs was investigated by EMSA. The mobility shift assays shown in Figure 3 indicate that both UVC and UVB increased the DNA binding capacity of p53 in nuclear extracts of irradiated fibroblasts, demonstrated by the formation of a DNA-protein complex (lanes 9 &11 and 13 &15, black arrow). The UVC and UVB-induced DNA-binding capacity was shown to be specific for p53, as the complex was supershifted with p53 antibody (lanes 12 and 16, asterisk), was outcompeted by excess unlabelled oligonucleotide (lanes 10 and 14) and was unreactive to a p21 oligonucleotide containing a mutated p53-binding site (lanes 11 and 15).

As shown in Figure 3, a UVA-inducible DNA-protein complex was observed following irradiation of fibroblasts at the 2 and 6 hour time points post insult but was undetectable by 12 hours. This transient UVA-induced DNA binding activity was capable of binding to the canonical DNA binding sequence but was not specific for p53 as the complex was not supershifted by the addition of a p53 antibody (lane 8), was outcompeted by an excess of both wild-type and mutant unlabelled probe (lanes 7 and 8 respectively). Therefore no detectable increase in DNA binding activity by p53 was observed after UVA at any of the time points investigated, suggesting that in response to UVA, p53 was not active as a transcription factor.

### The Induction of Apoptosis by UV Radiation

**Figure 4:**
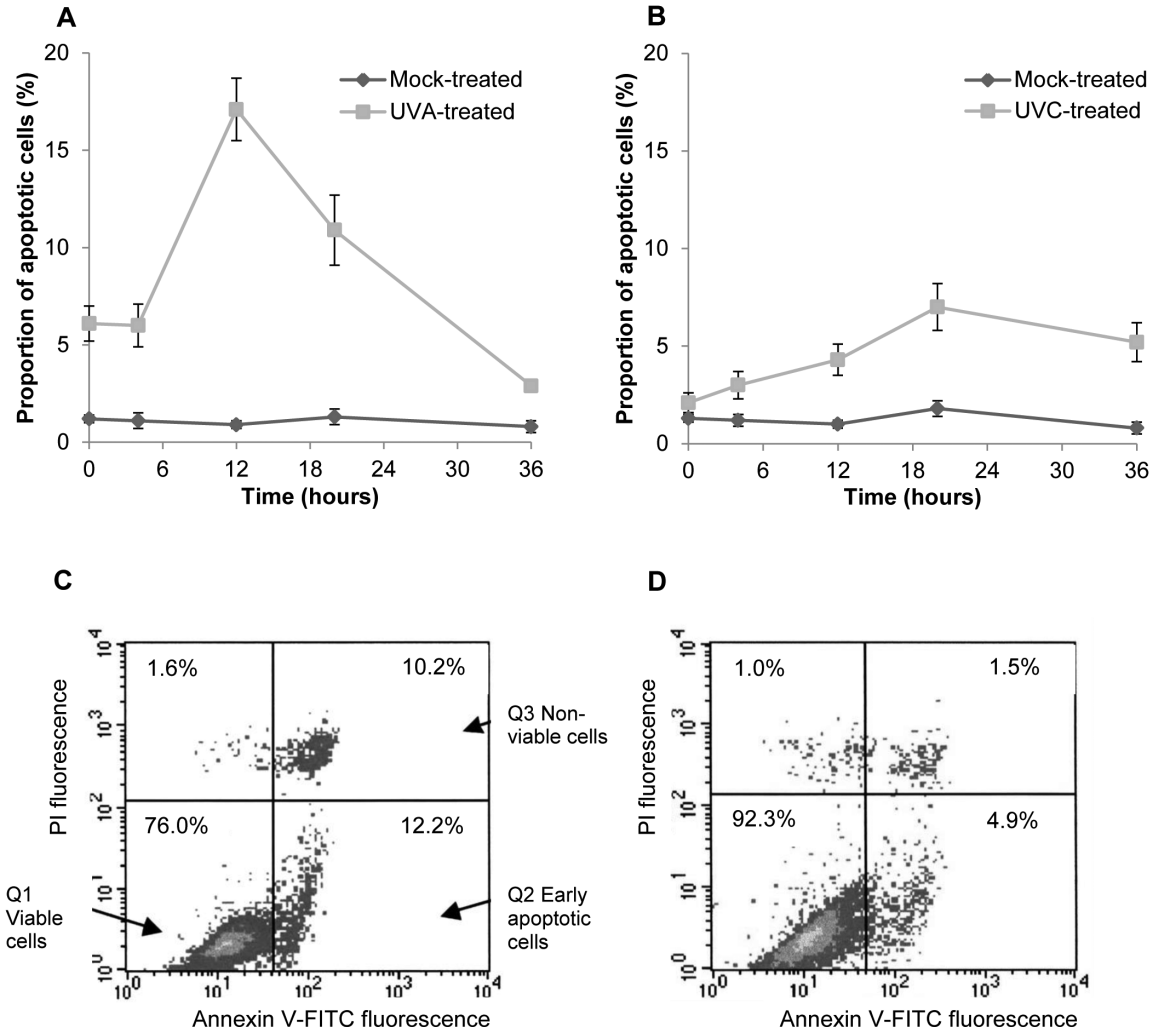
Induction of cell death in fibroblasts after exposure to ultraviolet radiation. Post-exposure time courses of induction of apoptosis in mouse fibroblasts exposed to **A** 1×10^5^ Jm^−2^ UVA and **B** 5 J m^−2^ UVC as determined by fluorescein isothiocyanate -annexin V/propidium iodide staining and fluorescence activated cell sorting flow cytometry. The percentages of annexin V-binding only cells are shown and each data point represents the mean of three independent experiments ± S.E.M. Representative density dot plots are shown for the respective peaks of apoptosis induction **C** 12 hours after UVA exposure and **D** 20 hours after UVC exposure. The lower left quadrant shows the viable cells, which exclude propidium iodide and are negative for fluorescein isothiocyanate -annexin V binding (Q1). The upper right quadrant (Q3) contains non-viable cells, positive for annexin V- fluorescein isothiocyanate binding and the uptake of propidium iodide. The lower right quadrant (Q2) represents the apoptotic cells, which are positive for annexin V- fluorescein isothiocyanate binding but exclude propidium iodide, demonstrating cytoplasmic membrane integrity. S.E.M = Standard error of the mean; UVA = ultraviolet A; UVC = ultraviolet C.

We sought to determine whether apoptosis plays a role in the cytotoxicity shown in Table 1. Cells were exposed to either 1×10^5^ J m^−2^ UVA or 5 J m^−2^ UVC. Apoptosis was quantified by flow cytometry of annexin V/PI stained cells or by determination of the sub G1 population. Figure 4 shows representative DNA histograms of mouse fibroblasts as observed 12 hours after irradiation with 100 k Jm^−2^ UVA (Figure 4C) and 20 hours post UVC exposure (Figure 4D). The time-course of events is presented in Figures 4A and B. Flow cytometric analysis of UVC irradiated MEFs revealed a statistically significant increase in the number of apoptotic cells 4 hours after treatment. Peak values of annexin V-FITC-positive cells were noted 20 hours after UVC exposure, moving towards control levels by 34 hours. Overall the level of UVC induced apoptosis was relatively low. In contrast to UVC, UVA induced immediate apoptosis shown by a significant increase in annexin binding cells, detected at the 0 hour time point in MEFs. The percentage of annexin positive cells continued to increase at a similar rate up to 4 hours after the initial insult. Peak values of annexin V-FITC positive cells were detected 12 hours after UVA exposure in cells, returning to control levels by 34 hours.

### Cell cycle delay after UV irradiation

By inducing a pause in progression through the cell cycle, the checkpoints are believed to provide additional time for repair of DNA damage and for inducing the transcription of genes required to facilitate repair or if damage is severe, for initiating an apoptotic response. Using an analysis of DNA profiles it is evident that unirradiated fibroblasts displayed a lag period of 6–12 hours before progressing into S phase of the cell cycle. After this time a synchronous wave of cells entered S from G1 and continued to traverse the cell cycle, entering G2/M after 18 hours. UVC irradiation of G1 synchronised cells induced a marked but transient G1 arrest such that 91.5±0.9% of these cells were arrested in G1 at 12 hours and 87.0±1.0% at 18 hours. UVA irradiation also produced a delay in entry to G1 with 84.6±0.9% of cells arrested in G1 at 12 hours although in this case recovery was more rapid with 24.4±3.9% of cells in G1 at the 18 hour time point.

**Figure 5:**
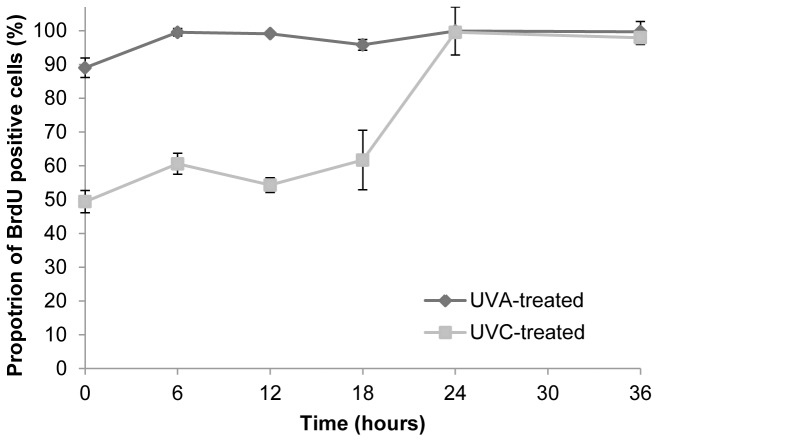
The progression of cells irradiated in S phase of the cell cycle. Cells were pulse-labelled with bromodeoxyuridine for 1 hour prior to sampling. The mean green fluorescence intensity of irradiated cells is shown as a percentage of the corresponding mock-irradiated cells at each time point following irradiation. Results shown are the mean ± S.E.M. of three independent experiments. S.E.M. = standard error of the mean; UVA = ultraviolet A; UVC = ultraviolet C.

Bivariate PI/BrdU analysis was performed to provide a more dynamic picture of the cell cycle post irradiation. Figure 5 shows that UVC exposure of cells resulted in a decreased BrdU uptake by S phase cells, indicative of a cessation of DNA synthesis. This was reflected by a decrease in the mean fluorescence intensity of cells stained with anti-BrdU-FITC relative to control mock-irradiated cells and persisted for 18 hours post exposure time. Synthesis was resumed by 36 hours as indicated by an increase in BrdU incorporation. An initial inhibition of BrdU uptake was observed in response to UVA exposure. This returned to control levels within 6 hours of UVA irradiation.

## Discussion

The data presented here demonstrate that the time course, extent and biological consequences of p53 induction are dependent on the wavelength of the UV to which mammalian cells are exposed. UVB and UVC irradiation of primary mouse fibroblasts triggered a persistent and dose dependent accumulation of p53 protein, in addition to increasing its capacity to bind to sequence specific DNA. The dose dependence of p53 accumulation in response to both UVB and UVC suggests that the degree of p53 induction may be directly related to the number of photoproducts in DNA. In contrast, at the equitoxic UVA doses investigated, no detectable increase in p53 protein levels or in the sequence-specific DNA-binding activity of p53 was detected in primary mouse fibroblasts.

Our observations are consistent with a recent study that showed that UVA was unable to induce Ser15 phosphorylation of p53 in primary human fibroblasts at equimutagenic doses to those at which UVB was able to induce a phosphorylation response [23]. Ser15 phosphorylation is one of a plethora of post-translational modifications that are associated with stabilisation and activation of p53. The data in the present study suggest that the inability of UVA to induce Ser15 phosphorylation, as observed by Runger and co-workers, results in an associated loss of p53 stabilisation and activation. Similar effects have been seen in other cell types; UV-irradiated melanocytes show an increase in overall p53 levels following UVB irradiation but not UVA [24].

Erythemal doses of UVA1 were unable to induce detectable Ser15 phosphorylation in skin biopsies taken from irradiated human subjects, and while levels of p53 itself did increase following irradiation this increase was far less than that induced by equivalent erythemal doses of UVB and solar-simulated radiation [25]. Other reports have also shown reduced p53 immunostaining in skin biopsies following *in vivo* erythemal doses of UVA compared to that seen with UVB [26], [27].

We have also examined the kinetics of p53 accumulation and find that it peaks between 12 and 18 hours post-irradiation with UVC. An increase in p53 protein levels is associated with the induction of cell cycle arrest and/or apoptosis. Indeed, following UVC exposure of G1 synchronised primary murine fibroblasts the results presented provide evidence of a pronounced delay in the onset of replication (Figure 5). The induction of both apoptosis and cell cycle arrest by UVC are associated with p53 perhaps by ATR kinase [28], [29] reviewed in [30]. Our data also show that induction of apoptosis shows similar kinetics to accumulation of p53 (Figure 4), supporting the importance of p53 in mediating UVC-induced apoptosis.

In marked contrast, we found that equitoxic doses of UVA that were unable to induce p53 phosphorylation caused only a transient delay in entry into S phase (Figure 5) and an earlier peak of apoptosis (Figure 4). We also find an increased number of cells in the upper right quadrant of the flow cytometric PI/FITC-Annexin V dot plot, which could correspond to late apoptotic or may be indicative of necrotic cells. Our data therefore show that following UVA-irradiation significant levels of cell death occur in a p53-independent fashion. Thus studies that use p53 status as a marker of damage in studies of photoprotection for example [31] may underestimate the damage done by UVA.

In conclusion, our findings demonstrate that an increase in p53 protein stability and sequence specific DNA binding is not a prerequisite for the induction of cell cycle checkpoints or apoptosis in cells following exposure to UV radiation in murine fibroblasts. In addition, the cellular response has been shown to be UV wavelength dependent. While it will be important to assess this in human epithelial cells, these findings have important implications regarding experimental photobiology; there is the need to exercise much greater caution when making assumptions about the contribution being made by different wavelengths of UV to specific cellular endpoints. We have shown that the extensively used “nonsolar” model mutagen (UVC; 254 nm) does not accurately replicate the effects of environmentally relevant UVA. Thus, great care needs to be taken when using molecular endpoints, such as p53, as biomarkers of UV exposure and the accumulation of p53 in response to mixed wavelengths of UV cannot be reliably used as an indicator of exposure or likely damage. This has both theoretical and practical implications for human protection against the harmful effects of cytotoxic agents, in particular when developing appropriate experimental methods and models for the investigation of skin cancer.
